# Chikungunya in an Infant: Diffuse Hyperpigmentation and the “Chik Sign”

**DOI:** 10.4269/ajtmh.20-1442

**Published:** 2021-07-06

**Authors:** Jami Rupa Ramani, Hima Gopinath, Prabhakaran Nagendran, Thirunavukkarasu Arun Babu

**Affiliations:** 1 Department of Dermatology, All India Institute of Medical Sciences, Mangalagiri, Andhra Pradesh, India;; 2 Department of Dermatology, All India Institute of Medical Sciences, Mangalagiri, Andhra Pradesh, India;; 3 Department of Dermatology, All India Institute of Medical Sciences, Mangalagiri, Andhra Pradesh, India;; 4 Department of Paediatrics, All India Institute of Medical Sciences, Mangalagiri, Andhra Pradesh, India

A 35-day-old male infant presented with generalized hyperpigmentation. Three weeks before presentation, the child had sustained a fever for 5 days. The parents had no history of fever. Pigmentation developed 8 days after the onset of fever. He had no history of skin lesions. On examination, the child was afebrile. There was generalized macular brown-to-black hyperpigmentation with interspersed patchy areas of normal skin. Accentuated pigmentation was observed on the nose (Chik sign) and lips (Figure 1). The palms and soles exhibited diffuse hyperpigmentation. The oral mucosa was normal. Based on the clinical presentation and endemicity of chikungunya in the region, a diagnosis of chikungunya pigmentation was considered. Immunoglobulin M (IgM) antibodies against the chikungunya virus were positive, thus confirming the diagnosis. The parents were reassured and the child was followed-up. Mildly reduced pigmentation and an increased area of normal skin were observed during follow-up 1 week later (Figure 2).

**Figure 1.**
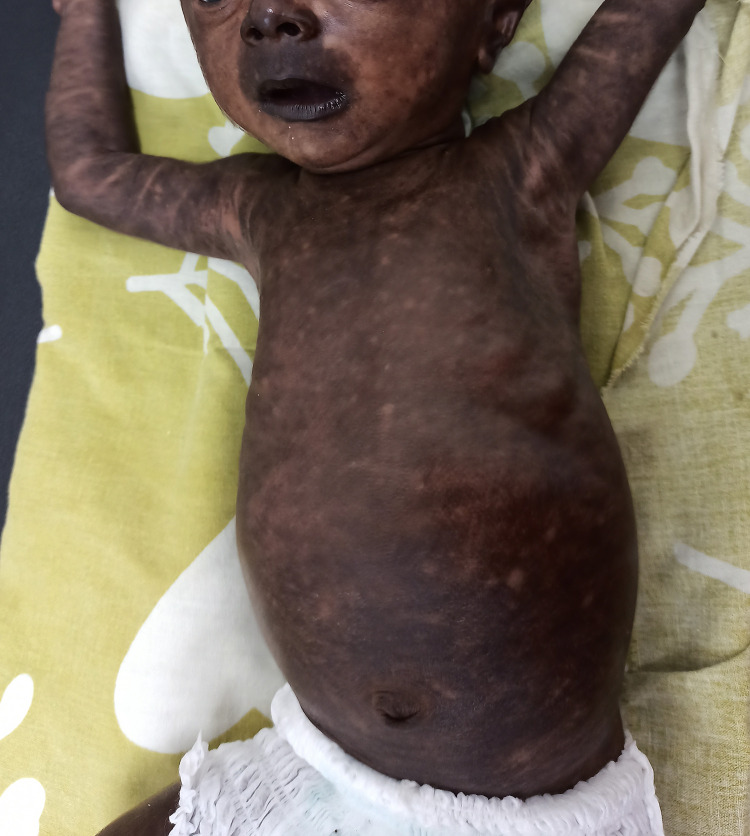
Generalized brown-to-black macular hyperpigmentation, “Chik sign,” and areas of spared normal skin. This figure appears in color at www.ajtmh.org.

**Figure 2.**
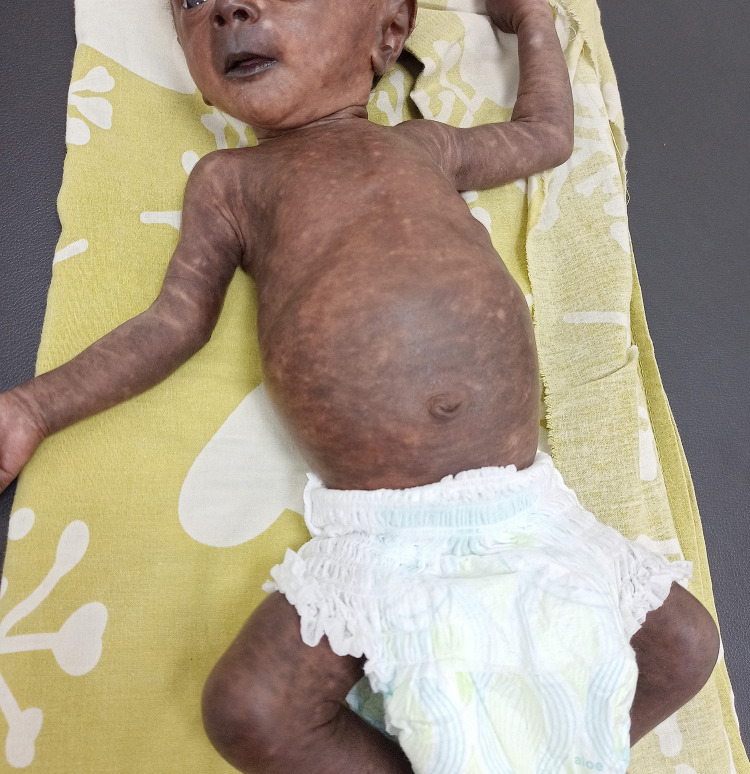
Slightly reduced pigmentation and increased areas of normal skin at the follow-up examination. This figure appears in color at www.ajtmh.org.

Chikungunya fever, an arboviral disease, is endemic in India, Southeast Asia, and Africa.^1^ Neonatal infection can occur through maternal–fetal transmission or through mosquito bites (*Aedes aegypti* and *Aedes albopictus*). Chikungunya fever presents with fever and polyarthralgia or polyarthritis. Cutaneous manifestations can be seen in 40% to 75% of cases.^2^ Chikungunya is an important cause of diffuse acquired pigmentation. Generalized pigmentation is commonly seen in infants, whereas pigmentation of the centrofacial area and neck is observed in adults. Brownish pigmentation of the tip of the nose (“Chik sign” or the “brownie nose appearance”) is a characteristic presentation of chikungunya.^1^ Flagellate pigmentation and mucosal pigmentation can also occur. Other cutaneous features include morbilliform rash, pigmentary changes, vesiculobullous lesions, urticarial lesions, vasculitis lesions, acrocyanotic lesions, purpuric lesions, erythema multiforme-like lesions, aphthous-like ulcers, lichenoid lesions, Stevens-Johnson syndrome, and toxic epidermal necrolysis-like lesions.^2^^,^^3^ The diagnosis can be confirmed by testing for IgM antibodies that develop within 1 week of symptoms.^1^ Our case highlights the importance of pigmentary changes in the diagnosis of chikungunya in endemic countries.

## Supplemental figure


Supplemental materials
