# Supplementary figures and images for: Chikungunya in an Infant: Diffuse Hyperpigmentation and the “Chik Sign”
Source: Am J Trop Med Hyg. 2021 Jul 6;105(3):547–8. doi: 10.4269/ajtmh.20-1442 (PMC8592348; doi:10.4269/ajtmh.20-1442)

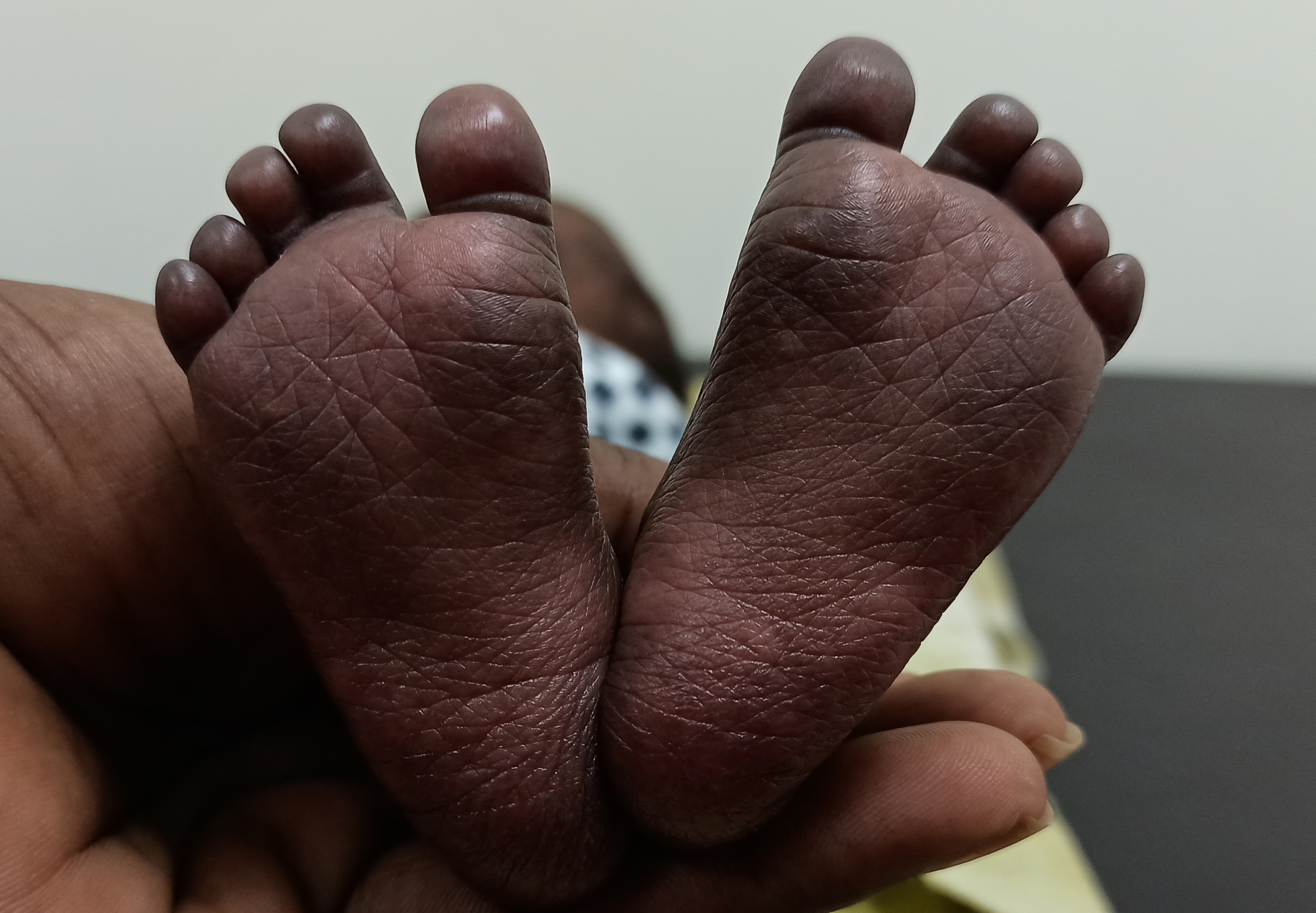

Supplement: Supplementary file 1 [file tpmd201442.SD1.jpg]
